# Impact of Nutrition Education on Nutrition Knowledge, Attitudes, Practices, and Immune-Related Nutrient Intake in People Living with HIV: A Randomized Controlled Trial

**DOI:** 10.3390/nu18111709

**Published:** 2026-05-27

**Authors:** Souheir M. Alia, Taoufik L. Zoubeidi, Habiba I. Ali

**Affiliations:** 1 Department of Nutrition and Health, College of Medicine and Health Sciences, United Arab Emirates University, Al Ain P.O. Box 15551, United Arab Emirates; 201570291@uaeu.ac.ae; 2 Department of Analytics in the Digital Era, College of Business and Economics, United Arab Emirates University, Al Ain P.O. Box 15551, United Arab Emirates; taoufikz22@gmail.com; 3 Interdisciplinary School of Health Sciences, Faculty of Health Sciences, University of Ottawa, Ottawa, ON K1N 6N5, Canada

**Keywords:** patients living with HIV, nutrition intervention, Health Belief Model, randomized controlled trial, United Arab Emirates

## Abstract

**Objective**: Nutrition is critical for people living with human immunodeficiency virus (PLHIV); nonetheless, nutritional interventions have not been conducted among PLHIV in the Middle East and North Africa region. This study evaluated the effects of a nutrition-related education intervention on total knowledge, attitude, and practice (KAP) scores and on the intake of immune-enhancing foods and nutrients among PLHIV. **Methods**: Sixty-three PLHIV were recruited from an outpatient HIV clinic in Dubai, United Arab Emirates, between August and November 2023 and randomly assigned to an intervention (*n* = 31) or control group (*n* = 32). The intervention group participated in an individualized, six-session nutrition education program based on the Health Belief Model, whereas the control group received usual care plus a nutrition education brochure on HIV nutrition and health. Data were collected at baseline and after the five-month intervention period using validated instruments assessing HIV-related nutrition knowledge, attitudes, and practices. A food frequency questionnaire and two non-consecutive 24 h dietary recalls were used to assess the intake of immune-enhancing nutrients. **Results**: Post-intervention KAP score distributions differed significantly between the control and intervention groups for knowledge, attitude, and practices (*p* < 0.001, 0.003, and 0.001, respectively). Immune-enhancing vitamin intake did not differ significantly between groups, except vitamin E, which increased in the intervention group (*p* = 0.042). **Conclusions**: The intervention improved participants’ nutrition-related KAP scores but did not increase the intake of immune-enhancing nutrients, except for vitamin E. Further studies are warranted to develop interventions that improve the intake of immune-enhancing nutrients.

## 1. Introduction

Human immunodeficiency virus (HIV) substantially compromises immune function, thereby making people living with HIV (PLHIV) more vulnerable to infections and illnesses [1]. One physiological consequence of HIV infection is unintended weight loss and muscle atrophy, negatively affecting general health, physical strength, and overall quality of life [2]. Adequate nutrition is therefore considered a key component of HIV/acquired immunodeficiency syndrome (AIDS) management. Specific nutrients, such as protein, vitamins A, C, and E, and zinc, support immune function and reduce susceptibility to infections [3]. Additionally, nutritional strategies can alleviate side effects of HIV medications, including nausea, diarrhea, and appetite loss, which often result in reduced nutrient intake and worsened nutritional status [2].

Although HIV/AIDS has received substantial global attention, research in the Middle East and North Africa (MENA) region addressing HIV-related nutritional deficiencies, nutritional status, and intervention strategies for PLHIV remains limited [4]. This gap is concerning because PLHIV across MENA face challenges beyond medical care, including malnutrition, limited nutritional awareness, low physical activity levels, lack of social and familial support, and persistent social stigma [4,5]. Without appropriate dietary intervention and ongoing support, these challenges can ultimately undermine the effectiveness of antiretroviral therapy (ART) and hinder recovery and quality of life [4,5].

Effective nutrition education programs help PLHIV mitigate medication-related side effects, improve dietary intake, and better manage disease-related complications. Furthermore, lifestyle-based approaches, particularly those grounded in behavior change theories such as the Health Belief Model (HBM), offer a promising framework for motivating sustained changes in health-related behaviors [6]. Given these needs and limited regional data, this study aims to provide insights into the implementation and evaluation of structured nutrition interventions tailored to the MENA context.

The Health Belief Model (HBM) is a well-known psychological framework that looks at how people think about their risk of getting sick, how bad it is, what advantages they think they will get, what barriers they think they will face, what cues they think would make them take action, and how confident they are in their ability to do something [7]. The model has been used a lot in health and lifestyle education programs because it helps people become more aware of health risks, encourages them to change their behavior, identifies perceived barriers, and helps them come up with personalized plans to encourage and maintain healthy behaviors over time through reminders, follow-ups, and reinforcement [8]. The HBM has been shown to help people living with HIV stick to their treatment plans, take part in preventive measures, and use healthcare services more often. Recent studies have also shown that it can help promote HIV testing, counseling, and peer-led preventive interventions [9,10]. The HBM was used in this study to help plan and carry out a structured nutrition education intervention aimed at improving the dietary knowledge, attitudes, practices, and behaviors of people living with HIV because it has a strong theoretical basis and has been shown to work in HIV-related health education [11].

This study sought to assess the efficacy of a six-session nutrition-oriented lifestyle intervention in enhancing nutrition-related knowledge, attitudes, and practices (KAP), as well as the consumption of immune-supportive nutrients, among individuals living with HIV (PLHIV) in the UAE. We hypothesized that the five-month intervention would result in significant improvements in KAP scores and nutritional intake relative to baseline.

## 2. Materials and Methods

### 2.1. Study Design and Participants

This interventional study was conducted among PLHIV attending an outpatient HIV clinic in Dubai, UAE. Between August and November 2023, 172 participants visiting the clinic were invited to participate; 63 (37%) consented and were enrolled. Eligibility was restricted to Dubai residents aged 18–60 years, excluding those with psychiatric illnesses or who were incarcerated. The 63 participants were randomized to either a control group (*n* = 32), which received usual care and a nutrition education brochure on HIV nutrition and health, or an intervention group (*n* = 31), which, in addition to receiving the nutrition education brochure, participated in an individualized six-session nutrition education program delivered over approximately 5 months.

Data were collected from all participants at baseline, pre-randomization, and post-intervention using validated instruments assessing nutrition KAP, and the intake of immune-enhancing nutrients. The primary outcomes were KAP scores, with anticipated increases of at least 1.71, 1.08, and 1.20 points in knowledge, attitude, and practice, respectively. Secondary outcomes included consumption of immune-enhancing nutrients and food groups, for which increased consumption was also expected due to the intervention.

The trial was registered at ClinicalTrials.gov (NCT06595225). https://clinicaltrials.gov/study/NCT06595225 (Date of registration: 17 August 2024). The CONSORT checklist is provided in the Supplementary Materials.

### 2.2. Sample Size Determination

The sample size was calculated for the primary outcome of the pre- and post-intervention difference in KAP scores. Using an independent-samples *t*-test with 90% power and a 0.05 significance level, the minimum sample size required to detect clinically significant between-group differences of 5, 2.9, and 1.1 in KAP scores, respectively, was determined. Based on standard deviations of 1.5, 1.5, and 1.2 for KAP, respectively [12], the resulting minimum sample sizes were 4, 7, and 27 participants per group, with the largest requirement (*n* = 27 per group) being selected. To account for a predicted 20% attrition rate, the sample size was increased to 33 participants per group, giving a predetermined sample size of 66.

To align with the gender distribution of the PLHIV population at the clinic (80% male and 20% female), stratified sampling was followed, with a planned composition of 26 males and seven females per group. Ultimately, 63 participants were included in the study.

### 2.3. Randomization

After recruitment and completion of baseline assessments, participants were randomized to either the intervention group (*n* = 31) or control group (*n* = 32) in a 1.03:1 allocation ratio. The random allocation sequence was generated by a statistician, using a computer-generated randomization program and stratified by gender and number of comorbidities (0, 1, or ≥2) to improve baseline comparability between groups. Randomization of the groups was performed only after baseline assessment to prevent allocation from influencing recruitment or baseline data collection. The principal investigator (PI) recruited and enrolled participants and assigned them to study groups according to the pre-generated randomization sequence. The allocation sequence was concealed until group assignment to minimize selection bias.

### 2.4. Intervention

The intervention, guided by the Health Belief Model (HBM) [13,14], lasted 5 months per participant. Both groups received the routine medical care and a basic dietary education manual. Moreover, participants randomized to the intervention group received individualized medical nutrition therapy delivered by a licensed dietitian over six sessions (40–45 min each), conducted either in person or online. Participants began the intervention after completing the baseline data collection. Each session included nutritional assessment, goal-setting, and education. The content of the sessions was developed by a multidisciplinary team, including dietitians/nutritionists, infectious disease physicians, a social worker, and a nurse, to ensure relevance for people living with HIV.

The sessions addressed several critical topics, including the risks associated with HIV-related nutrition, the necessity for early nutritional intervention, the difficulties of maintaining a healthy diet and lifestyle modifications, practical strategies for incorporating nutritious foods and exercise, nutrients that enhance immune function, the MyPlate dietary guidelines, and the management of nutrition in the context of comorbidities such as diabetes and dyslipidemia. The educational sessions were guided by the HBM constructs: perceived susceptibility (“nutritional risks associated with HIV”), perceived benefits (“importance of early nutrition intervention”), perceived barriers (“weighing out our options”), and cues to action (“ways of incorporating a healthy diet and lifestyle”), followed by individualized counseling and reinforcement sessions. Intervention participants received multimedia educational materials, written handouts, and weekly WhatsApp reminders to assist them in adhering to their objectives and enhance their nutritional knowledge, attitudes, and behaviors.

All participants in the intervention group completed all six planned nutrition education sessions. Sessions were delivered online; one participant attended two sessions in person due to individual circumstances. Weekly WhatsApp reminders were standardized and delivered using a consistent format throughout the intervention period. Intervention fidelity was ensured by closely adhering to the predefined lesson plans, educational materials, and intervention objectives for all sessions. Slight individual modifications were integrated when needed to address participants’ specific comorbidities and health-related concerns, while maintaining the overall structure and content of the intervention.

### 2.5. Evaluation of Intervention Efficacy

Intervention effectiveness was assessed by comparing baseline-to-post-intervention changes in immune-enhancing food and nutrient consumption and nutritional KAP between control and intervention groups. Baseline-to-post-intervention measurement changes were analyzed using appropriate statistical techniques for both within-group (intra-) and between-group (inter-) comparisons. Within-group comparisons assessed subject-level changes from baseline to post-intervention in each treatment group. Conversely, between-group comparisons evaluated the magnitude of changes in outcome variables across the two groups (control vs. intervention) while controlling for confounders.

### 2.6. Data Collection

Data were collected at the communicable disease outpatient clinic of Rashid Hospital, Dubai, UAE, specifically the HIV clinic. Baseline study data collection began on 28 August 2023, and concluded on 28 November 2023. Nonetheless, the interventional study began on 28 August 2023, and concluded on 30 April 2024. Baseline data collection and intervention implementation occurred concurrently rather than sequentially. As participants completed the baseline assessment and were randomized, those allocated to the intervention group immediately started the nutrition education intervention. Therefore, intervention activities commenced during the ongoing baseline recruitment and data collection period and continued on a rolling basis until 30 April 2024.

#### 2.6.1. Knowledge, Attitude, and Practices

The RCT utilized the same validated KAP questionnaire previously described in the cross-sectional phase of this project [15]. The instrument assesses knowledge (0–14), attitude (14–42), and practice (0–13), with total scores ranging from 13 to 69 and categorized as poor (13–32), average (33–51), and good (52–69).

#### 2.6.2. Dietary Intake Data

##### Twenty-Four-Hour Dietary Recall

Twenty-four-hour dietary recalls were conducted on non-consecutive days (one weekday and one weekend day), both at baseline and post-intervention. Dietary intake was assessed for total calories and protein and for nutrients with immune-supportive properties (vitamin A, vitamin C, vitamin E, vitamin D, selenium, zinc, omega-3 and omega-6 fatty acids, and iron). To improve accuracy, the dietary recall process incorporated portion-size aids (e.g., standard household measures), prompting techniques (time-interval prompts and a checklist of commonly forgotten foods), and probing questions regarding cooking methods. Diet recalls were analyzed using ESHA Food Processor SQL (ESHA Research, Salem, OR, USA, version 11.3).

##### Qualitative Food Frequency Questionnaire

A panel of seven nutritionists developed a qualitative FFQ. A pre-final version, formulated from the literature and expert opinion, was pilot-tested in 10 PLHIV from the target population; participants completed the FFQ and were interviewed to assess question clarity and acceptability [16]. All participants reported that the questions were sufficiently clear to understand and answer. Comprehensibility was further assessed by 10 panelists using Lawshe’s CVR [17,18].

The final FFQ version comprised 10 food categories recognized for immune-enhancing properties: fruits, vegetables, herbs, probiotic-rich foods, whole grains, nuts, seeds, animal-based proteins, plant-based proteins, and plant oils rich in monounsaturated (MUFA) and polyunsaturated fatty acids. Consumption frequency was recorded monthly, weekly, and daily, using diverse intervals per day (1, 2–3, 4–5, 6+/day), per week (1, 2–4, 5–6/week), or per month (never or 1, 1–3/month). The FFQ typically required 5–10 min to complete [19].

### 2.7. Anthropometrics

Weight, height, and body mass index were collected from the patients’ medical files at baseline and post-intervention.

### 2.8. Statistical Analysis

Data were analyzed using SPSS v28. Descriptive statistics (means and standard deviations or counts and percentages) were used to summarize data. Analyses included within- and between-group comparisons. Within-group comparisons examined changes in primary outcome variables from baseline to post-intervention within each group, assessing subject-level change over time. Conversely, between-group comparisons evaluated the magnitude of change in outcome variables between the two groups (control vs. intervention) while controlling for confounders.

For within-group comparisons, baseline-to-post-intervention changes within each group were assessed using paired *t*-tests for continuous variables and Pearson’s chi-square or Fisher’s exact tests for categorical variables. Fisher’s exact test was used for 2 × 2 tables. Cohen’s d was used to measure the effect size in the pairwise comparisons of KAP (pre vs. post values) within each group [20].

Post-intervention group effects on continuous outcomes were compared using linear regression adjusted for baseline outcome values, educational level, and marital status. The reference categories of the latter two predictors were “post-secondary education” and “neither single nor married”, respectively. A similar approach was applied to categorical outcomes using logistic or multinomial regression. Cohen’s f^2^ was used to assess the effect size of group intervention on the post-intervention values of the primary outcomes (KAP and immune-enhancing nutrients) while controlling for baseline values and covariates. Mean changes in quantitative variables (e.g., immune-enhancing nutrients and BMI) from baseline to post-intervention were compared between the control and intervention groups using an independent-samples *t*-test. When data were not normally distributed, the Mann–Whitney U test was used rather than the independent-samples *t*-test. Data normality was assessed using the Shapiro–Wilk test, and variance equality was assessed using Levene’s test. A *p*-value < 0.05 was considered statistically significant.

Both per-protocol (PP) and intention-to-treat (ITT) analyses were conducted to assess the sensitivity of findings to protocol deviations. The only non-adherence to the study protocol was loss to follow-up among 17 participants (10 in the control and 7 in the intervention group) out of 63. Missing post-intervention primary outcome values for these dropouts were estimated using multiple imputation. When imputing missing values for a KAP variable (e.g., knowledge), pre-intervention KAP values, post-intervention values of other KAP variables (e.g., practices, attitude, and total KAP), and marital and education status were used. The latter two variables were the only demographic variables that differed between the two groups.

Missing post-intervention values for immune-enhancing nutrients (vitamin A, C, E, D, selenium, iron, zinc, and protein) were estimated by multiple imputation using pre-intervention KAP values, marital and educational status, and pre- and post-intervention intake of all immune-enhancing nutrients. Demographic characteristics of dropouts and non-dropouts were compared to assess whether they represented distinct subpopulations.

### 2.9. Ethical Approval

Ethical approval for the trial was obtained from the Research Ethics Committee of Mohammed Bin Rashid University of Medicine and Health Sciences and the Dubai Students’ Research Ethics Committee (MBRU IRB-2023–72). All participants were informed about the study objectives, the voluntary nature of participation, and data confidentiality. Written and verbal informed consent was obtained before enrollment.

## 3. Results

**Figure 1:**
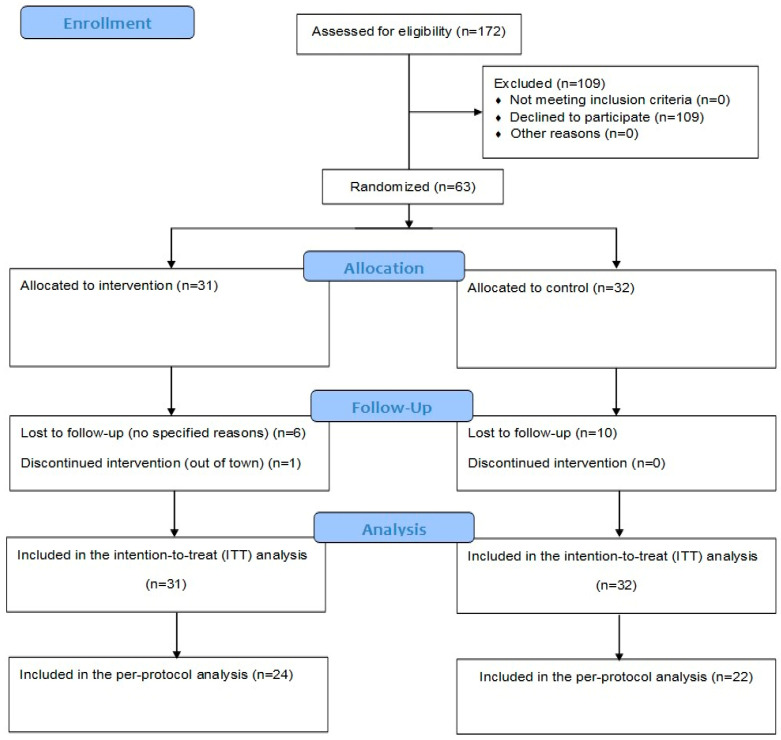
CONSORT diagram.

The participants’ flow in this randomized controlled trial, from enrolment to analysis, is presented in the CONSORT diagram (Figure 1).

Participants were equally allocated to the control and intervention groups (32 and 31, respectively). Ten (31.3%) and seven (22.6%) participants in the control and intervention groups dropped out of the study before the five-month endpoint. Dropout rates did not differ significantly between groups (Pearson’s chi-square test; *p* = 0.438). No significant differences were observed between dropouts and non-dropouts within either group regarding age, income, and education. However, gender distributions differed significantly between dropouts and non-dropouts in the control group (*p* = 0.03), but not in the intervention group.

### 3.1. Demographics

Educational level and marital status differed significantly at baseline (Table 1). Because these variables may influence nutrition knowledge, dietary practices, and response to nutrition education, they were included as covariates in the adjusted between-group analyses. Gender distribution was similar between groups, with males comprising the majority in both the control (72.7%) and intervention (81.8%) arms. Similarly, income levels were comparable: approximately one-third of control participants were in the lowest (<7000 AED/month) and highest (≥15,000 AED/month) income brackets, whereas 43.6% of the intervention group reported incomes ≥ 15,000 AED/month. These differences were not statistically significant.

Gender distribution and income level did not differ significantly between groups. Nonetheless, a significantly higher proportion of participants in the intervention arm had less than a high school education than those in the control group (34.5% vs. 12.7%; *p* = 0.026).

### 3.2. Knowledge, Attitude, and Practices

Participants’ KAP scores were assessed at baseline and post-intervention. A sensitivity analysis compared PP and ITT estimates of KAP scores. Under the ITT approach, missing post-intervention KAP values for participants who dropped out were estimated using multiple imputation. Both ITT and PP analyses demonstrated significant within- and between-group differences for the intervention group (Table 2).

Mean differences across groups were significant in both PP and ITT analyses (*p* < 0.05) for KAP and total KAP score (Table 2). Moreover, after adjustment for confounding variables, between-group differences remained significant for both individual KAP subcomponents and total KAP score in PP and ITT analyses (*p* < 0.05; Table 2).

Table 2 presents the findings from the PP or ITT analyses and shows significantly greater improvements in the intervention group across all KAP domains compared with the control group. Knowledge scores rose by 53% and 12% in the intervention and control groups, respectively (mean score change: 4.48 vs. 1.02), indicating a substantial post-intervention improvement in understanding. Attitude scores also exhibited a positive shift, with a 5% increase in the intervention group compared with no change in the control group (2.1 vs. 0.15), reflecting more favorable perceptions and greater openness toward the targeted behaviors.

Behavioral outcomes followed a similar pattern: the intervention group demonstrated a 26% improvement, whereas the control group showed a 4% decline (2.32 vs. −0.33), suggesting that the intervention effectively promoted favorable behavioral change. Overall, the total KAP score rose by 16% in the intervention group, compared with only 1% in the control group (8.9 vs. 0.86). Together, these differences indicate that the intervention led to statistically significant and meaningful improvements in participants’ KAP.

Moreover, based on Cohen’s f^2^ interpretation of effect size, the intervention demonstrated a large effect on nutrition-related knowledge (f^2^ = 0.466, 95% confidence interval = (0.12, 1.04)), practices (f^2^ = 0.363, 95% CI = (0.07, 0.87)), and total KAP score (f^2^ = 0.597, 95% CI = (0.18, 1.25)). In contrast, a medium effect was observed for nutrition-related attitudes (f^2^ = 0.165, 95% CI = (0.01, 0.52)).

### 3.3. Intake of Immune-Enhancing Nutrients

As presented in Table 3, most dietary sources of immune-enhancing nutrients showed no statistically significant post-intervention differences between the control and intervention groups after adjustment for baseline values, educational level, and marital status, the only two demographic variables that differed between groups. Nonetheless, several within-group improvements were observed. Both groups demonstrated a significant increase in MUFA intake, with daily consumption rising from 25% to 60.9% in the control group (*p* = 0.005) and increasing by 31% in the intervention group (*p* = 0.003). Collectively, these findings suggest a positive shift in fat quality across both arms, although post-intervention MUFA intake did not differ significantly between groups.

The intervention group demonstrated a significant increase in weekly wholegrain consumption, rising from 29% to 45.8% (*p* = 0.038), whereas the control group showed only a small, non-significant increase (25% to 30%). This indicates that the intervention encouraged healthier carbohydrate choices. Although not statistically significant, the daily intake of polyunsaturated fats demonstrated a favorable upward trend in the intervention group, increasing by 21.6% (*p* = 0.063) compared with 11.3% in the control group.

Regarding protein sources, animal-based protein intake rose significantly within the intervention group (*p* = 0.011); 21.7% consumed it four or more times per day post-intervention, compared with none in the control group. Other dietary components, including fruits, vegetables, nuts, seeds, and probiotics, exhibited non-significant improvements in intake frequency and regularity. Therefore, although overall between-group differences were not statistically significant, the intervention group demonstrated meaningful within-group improvements in wholegrain, MUFA, and animal-based protein consumption patterns, reflecting partial success in promoting healthier dietary habits.

As presented in Table 4, no statistically significant between-group differences were observed in post-intervention intake of immune-enhancing nutrients, except for vitamin E (*p* = 0.042). Vitamin E intake in the intervention group increased from 0.5 ± 0.76 mg to 0.8 ± 0.91 mg, whereas intake in the control group declined from 1 ± 1.33 mg to 0.5 ± 0.59 mg. Nonetheless, both groups remained below the recommended dietary allowance for vitamin E: 15 mg/day for adults (including pregnant women), 19 mg/day for lactating women, and 4–11 mg/day for children. Other nutrients, including iron, vitamins A, C, and D3, zinc, selenium, and omega-3 and omega-6 fatty acids, showed only minor, non-significant fluctuations from baseline to post-intervention.

Additionally, based on Cohen’s f^2^ interpretation of effect size, most nutrient intake outcomes demonstrated negligible or no meaningful intervention effect. Vitamin E intake showed the highest observed effect size among the assessed nutrients (f^2^ = 0.03), representing a small effect according to Cohen’s criteria. These findings suggest that although the intervention substantially improved nutrition-related knowledge, attitudes, and practices, its effect on actual nutrient intake was comparatively limited over the intervention period.

Macronutrient and energy intake remained stable in both groups. Protein intake increased minimally in the control group (100 ± 46.88 g to 103 ± 50.48 g) and declined from 105 ± 52.31 g to 101 ± 53.05 g in the intervention group. Similarly, total energy intake rose from 2131 ± 929.63 kcal to 2379 ± 970.95 kcal and decreased from 2210 ± 1351.68 kcal to 2069 ± 965.43 kcal in the control and intervention groups, respectively. Calcium intake increased slightly in both groups, from 561 ± 307.75 mg to 597 ± 361.83 mg and from 465 ± 311.06 mg to 583 ± 383.36 mg in the control and intervention groups, respectively. These changes were not statistically significant.

A sensitivity analysis comparing the PP and ITT models, controlling for confounders such as educational level and marital status, confirmed the robustness of these findings. Both analyses demonstrated consistent results across nutrients, with vitamin E remaining the only nutrient reaching statistical significance (PP: *p* = 0.002; ITT: *p* = 0.042). Thus, whereas the intervention did not produce broad dietary changes across most immune- enhancing nutrients, it was accompanied by a meaningful improvement in vitamin E intake in the intervention group. Although this effect was insufficient to achieve recommended dietary allowance levels, it supports the potential of targeted interventions to positively influence specific nutrient intakes.

## 4. Discussion

Guided by the Health Belief Model (HBM), this study developed and delivered a structured, six-session nutrition education intervention for people living with HIV (PLHIV) and evaluated its impact on nutrition-related knowledge, attitudes, and practices (KAP), as well as dietary intake, over five months. The intervention resulted in a significant improvement in total KAP scores among PLHIV in the intervention group. In contrast, no change was observed in the control group, supporting the effectiveness of the structured nutrition education program.

There were significant between-group improvements in the primary outcome measures, with the intervention group demonstrating greater increases in nutrition-related knowledge, attitudes, practices, and overall KAP scores compared with the control group. Moderate-to-large intervention effects were present across nutrition-related outcomes among PLHIV. According to Cohen’s f^2^ interpretation, large effect sizes were observed for knowledge, practices, and overall KAP scores, suggesting that the structured Health Belief Model-based nutrition education intervention had a substantial impact on participants’ nutrition-related understanding and behaviors. The effect size for attitudes was comparatively smaller, although still within the medium range, which may reflect the greater complexity and time required for attitudinal change compared with knowledge acquisition and behavioral modification. For the secondary outcomes, a significant between-group increase was observed only for the consumption of vitamin E-rich foods, favoring the intervention group. Moreover, within-group analyses showed significant improvements in the intervention group in the consumption of healthy dietary components, including protein, whole grains, and monounsaturated fatty acids (MUFAs). Overall, these findings support the effectiveness of individualized nutrition education and reinforcement strategies in improving nutrition-related outcomes among PLHIV in the UAE.

The results of the present study help address a substantial gap in nutrition-focused HIV interventions in the MENA region, where few programs have specifically targeted the KAP of PLHIV [21,22]. Consistent with global evidence, structured, theory-based nutrition education has been shown to positively influence dietary behaviors and nutrition knowledge in PLHIV [12,23,24,25]. Similar associations between improved dietary practices and enhanced nutrition knowledge have been reported in diverse settings, including Kenya and Ghana [26,27], India, Nigeria, and Uganda [28,29,30].

Importantly, unlike many interventions conducted in resource-constrained settings, where improvements in knowledge do not always translate into measurable dietary change because of socioeconomic barriers [29,30], the present intervention resulted in significant improvement of vitamin E consumption between groups as well as improvements within the intervention group in protein, wholegrain, and MUFA intake. These findings suggest that structured, culturally tailored nutrition education grounded in behavioral theory can facilitate meaningful dietary improvements when implemented within supportive healthcare systems.

Nonetheless, despite targeted education, persistent dietary deficiencies in calcium [31], zinc [32], and vitamin D were observed, reflecting challenges documented in other low- and middle-income settings [33]. Factors such as food insecurity, affordability constraints, limited dietary diversity, and cultural dietary patterns have been identified as key barriers to achieving optimal nutrient intake among PLHIV populations. Similarly, the lack of significant improvements in fruit, vegetable, probiotic, and polyunsaturated fat consumption aligns with previous research conducted in resource-limited environments, where education alone is insufficient to achieve full nutrient adequacy [34,35]. These findings highlight the limitations of education-only approaches and underscore the importance of addressing structural and environmental barriers to nutrition.

Moreover, previous research suggests that educational interventions alone may be insufficient to address the inadequate intake of vitamin D, zinc, and calcium, particularly in settings where food access, affordability, and broader nutritional challenges remain significant concerns, as similarly reported in Ghana, Kenya, and India [26,27,28]. These findings reinforce the need for comprehensive interventions that combine education with supplementation, food fortification, and improved access to nutrient-rich foods [34,35].

In the UAE context, nutrition education should remain an integral component of HIV care, complementing national public health initiatives. Although PLHIV in the Middle East continue to face stigma, fragmented healthcare services, and limited access to HIV-specific nutrition information, UAE national frameworks such as the Ministry of Health and Prevention’s National Nutrition Guideline provide a strong foundation for integrating targeted nutrition support [36]. Local evidence from Dubai further supports this approach, demonstrating that culturally adapted, HBM-based nutrition education programs can significantly improve knowledge, attitudes, and dietary behaviors among PLHIV [37]. These findings are particularly important given the continued prevalence of micronutrient imbalances and obesity, even in high-income settings such as the UAE [38,39,40].

Overall, optimal nutrition remains fundamental to comprehensive HIV care, supporting immune function, reducing opportunistic infections, improving adherence to antiretroviral therapy, and enhancing quality of life [41,42,43]. Nonetheless, the persistence of certain insufficient dietary sources highlights the need for integrated, multisectoral strategies. In the UAE, combining sustained nutrition education with supplementation programs, food fortification policies, and improved access to nutrient-dense foods may provide a comprehensive and sustainable approach to addressing nutritional gaps, strengthening clinical outcomes, and promoting long-term health and well-being among PLHIV.

### Strengths and Limitations

This study has several strengths. First, it is the first nutrition-education intervention for PLHIV in the UAE and the Arab Gulf region. Second, the educational program was guided by the HBM and adapted to the UAE cultural context. Additionally, key advantages included emphasis on non-marginalized populations in the MENA and evidence of improved health outcomes.

One limitation was the low response rate, likely attributable to HIV-associated stigma. Another limitation was the small sample size. Conclusions regarding long-term sustainability were limited because of the short follow-up period. Nutrient intake was assessed by self-report. However, supplement use and biochemical validation were not considered. Finally, adherence tracking beyond the five months is needed to assess whether effects are maintained over a longer timeframe.

Another limitation was the baseline differences in the control and intervention groups in educational attainment and marital status. Although adjusted analyses were performed to control for these potential confounders, these variables may have influenced the receptiveness of the participants to the nutrition education intervention. Higher educational levels might improve understanding and application of health information and marital status might influence social support, motivation and compliance with recommendations for lifestyle change. Therefore, results should be interpreted in the light of these differences in baseline. We adjusted the analyses to reduce these potential confounders, but these characteristics may have influenced participants’ responsiveness to the nutrition education intervention.

Moreover, the assessment of dietary intake was based on self-reported methods, such as 24 h dietary recalls and FFQ, which are prone to recall bias, inaccuracies in reporting, and social desirability bias. Participants in the intervention group may have been more aware of the recommended dietary behaviors after the nutrition education sessions, which may have influenced their responses and resulted in overreporting of healthier food choices or underreporting of less desirable dietary practices. Although standardized dietary assessment procedures were used to minimize these limitations, the possibility of reporting bias cannot be completely ruled out when interpreting the dietary intake results. Nutrition education sessions may have increased participants’ awareness of recommended dietary behaviors, which may have influenced their responses and resulted in the overreporting of healthier food choices or the underreporting of less desirable dietary practices. Although standardized dietary assessment procedures were used to minimize these limitations, the possibility of reporting bias cannot be completely eliminated in interpreting the dietary intake findings.

Finally, the trial was retrospectively registered at ClinicalTrials.gov (Identifier: NCT06595225) after the study concluded. However, there were no changes in the study protocol, eligibility criteria, intervention procedures, and planned outcomes throughout the study period.

## 5. Conclusions

This study emphasizes the role of food and lifestyle interventions in the UAE for improving the health outcomes of PLHIV, which may have implications for the broader population of PLHIV in the MENA region. This study assessed the impact of six nutrition and lifestyle educational intervention sessions on PLHIV’s nutritional KAP and food consumption. The intervention group, which received individualized HBM-based training, demonstrated improved KAP scores and vitamin E intake; however, the intake of other nutrients did not significantly improve. Thus, despite significant improvements in nutrition-related knowledge, attitudes, and practices, the intervention had limited effects on most nutrient intake outcomes, suggesting that behavioral improvements may not immediately translate into measurable changes in dietary intake and may require longer intervention durations and continued reinforcement strategies. These findings highlight the importance of targeted interventions to improve nutrition education and related health behaviors among PLHIV. Additional research is warranted to further address these essential components of HIV care.

To better understand how dietary interventions influence HIV outcomes over time, future longitudinal studies should include culturally adapted interventions tailored to the MENA region, with particular focus on micronutrient deficiencies such as zinc, selenium, and vitamin D, as well as the role of physical activity in HIV outcomes. Additionally, investigating digital or remote interventions may improve healthcare accessibility in the region, while exploring demographic differences in KAP may further support optimization of HIV care.

## Figures and Tables

**Table 1:** Demographic distribution of the participants.

Age (Mean ± SD)		Group		
		Control	Intervention	*p*-Value
		38.93 (12.26)	40.36 (10.89)	0.517
Gender *n* (%)	Male	23 (72.7)	25 (81.8)	0.182
	Female	9 (27.3)	6 (18.2)	
Income level *n* (%)	<7000 AED/month	9 (27.3)	7 (23.6)	0.233
	<7000 & ≥15,000 AED/month	10 (32.7)	7 (23.6)	
	>15,000 AED/month	8 (25.5)	14 (43.6)	
	Others	5 (14.5)	3 (9.1)	
Educational Level *n* (%)	Less than high school	4 (12.7)	11 (34.5)	0.026
	High school	16 (50.9)	12 (40.0)	
	Post-secondary	12 (36.4)	8 (25.5)	
Marital Status *n* (%)	Single	17 (54.5)	11 (34.5)	0.005
	Married	13 (40.0)	12 (38.2)	
	Others	2 (5.5)	8 (27.3)	
Duration of Illness (Mean ± SD)		8.56 (6.63)	8.27 (7.27)	0.827
BMI group/pre-intervention *n* (%)	Underweight	2 (6.7)	1 (3.2)	0.144
	Normal weight	5 (16.7)	12 (38.7)	
	Overweight	16 (53.3)	9 (29.0)	
	Obese	7 (23.3)	9 (29.0)	
BMI group/post-intervention *n* (%)	Underweight	1 (4.3)	1 (4.3)	1.00
	Normal weight	7 (30.4)	7 (30.4)	
	Overweight	9 (39.1)	9 (39.1)	
	Obese	6 (26.1)	6 (26.1)	
BMI differences (Mean ± SD)	Underweight	27.4 ± 5.2	27.1 ± 6.1	0.521
	Normal weight	27.2 ± 5.4	27.0 ± 6.1	0.869
	Overweight	−0.75 ± 3.7	0.45 ± 4.1	0.196
	Obese	23 (72.7)	25 (81.8)	0.182

Fisher’s exact test was used for gender (*p*-value < 0.05 is considered statistically significant). Pearson’s chi-square was used for income, educational level, BMI categories, and marital status (*p*-value < 0.05 is considered statistically significant). An independent-sample Mann–Whitney U test was used for BMI differences (*p*-value < 0.05 is considered statistically significant). An independent-samples *t*-test was used for age and duration of illness (*p*-value < 0.05 is considered statistically significant). BMI; body mass index, *n*; sample size, SD; standard deviation.

**Table 2:** Impact of nutrition education on KAP scores breakdown.

		Total (Mean ± SD)		*p*-Value * (PP)	Effect Size ^§^ (PP)	*p*-Value * (ITT)	Effect Size ^§^ (ITT)
Section	Period	Control (*n* = 32)	Intervention (*n* = 31)				
Knowledge (PP)	Baseline	8.41 ± 2.60	8.48 ± 3.09	<0.001	0.466	<0.001	0.342
	PI	9.43 ± 1.78	12.96 ± 1.49				
Knowledge (ITT)	Baseline	9.04 ± 2.45	8.96 ± 2.90				
	PI	9.43 ± 1.80	12.96 ± 1.50				
Gain in Score		1.02	4.48				
Quantum of Improvement		1.12	1.53				
Percentage of Change (%)		12	53				
*p*-value ^+^ (PP)		0.534	<0.001				
Effect size ^#^ (PP)		0.132	1.566				
*p*-value ^+^ (ITT)		0.128	<0.001				
Effect size ^#^ (ITT)		0.347	0.561				
Attitude (PP)	Baseline	40.50 ± 1.48	39.90 ± 3.07	0.013	0.165	0.003	0.131
	PI	40.65 ± 1.11	42 ± 0.00				
Attitude (ITT)	Baseline	40.48 ± 1.65	39.80 ± 3.31				
	PI	40.65 ± 1.11	42 ± 0.00				
Quantum of Improvement		1	1.05				
Percentage of Change (%)		0	5				
*p*-value ^+^ (PP)		0.676	0.003				
Effect size ^#^ (PP)		0.088	0.679				
*p*-value ^+^ (ITT)		0.504	<0.001				
Effect size ^#^ (ITT)		0.211	0.612				
Practices (PP)	Baseline	8.94 ± 2.35	8.97 ± 2.73	<0.001	0.363	0.001	0.205
	PI	8.61 ± 1.70	11.29 ± 1.57				
Practices (ITT)	Baseline	9.04 ± 2.34	8.42 ± 2.80				
	PI	8.61 ± 1.70	11.30 ± 1.57				
Gain in Score		−0.33	2.32				
Quantum of Improvement		0.96	1.26				
Percentage of Change (%)		−4	26				
*p*-value ^+^ (PP)		0.436	<0.001				
Effect size ^#^ (PP)		−0.166	0.974				
*p*-value ^+^ (ITT)		0.815	<0.001				
Effect size ^#^ (ITT)		0.019	0.613				
Total KAP (PP)	Baseline	57.84 ± 3.79	57.35 ± 6.72	<0.001	0.597	<0.001	0.453
	PI	58.70 ± 2.36	66.25 ± 2.29				
Total KAP (ITT)	Baseline	58.61 ± 3.76	57.13 ± 7.06				
	PI	58.70 ± 2.40	66.30 ± 2.30				
Gain in Score		0.86	8.9				
Quantum of Improvement		1.01	1.16				
Percentage of Change (%)		1	16				
*p*-value ^+^ (PP)		0.933	<0.001				
Effect size ^#^ (PP)		0.018	1.342				
*p*-value ^+^ (ITT)		0.334	<0.001				
Effect size ^#^ (ITT)		0.256	1.027				

*: Linear regression test was used to determine differences across groups pre- versus post-intervention (*p*-value < 0.05 is considered statistically significant) while controlling for the two significant confounding factors (educational level and marital status). ^+^: A paired sample *t*-test was used to determine differences within a single group before and after the intervention. ^#^: Cohen’s d measure of effect size. Interpretation: 0.20 (small effect), 0.50 (medium), 0.80 (large) [20]. ^§^: Cohen’s f-square measure of effect size. Interpretation: 0.02 (small effect), 0.15 (medium), 0.35 (large) [20]. KAP; knowledge, attitude, and practices, ITT; intention-to-treat, *n*; sample size, SD; standard deviation, PI; post-intervention, sample size differed based on method of analysis, 63 participants for ITT analysis and 46 for PP analysis. The values of gain in score (GS), quantum improvement (QU), and % change (%C) are based on the per-protocol values. The formulas that were used to calculate gain in score (GS), percentage of change (%C), and quantum of improvement (QI) were: GS = Values at post-intervention − value at baseline. %C = 100 × (Value at post-intervention. − value at baseline)/value at baseline.

**Table 3:** Consumption of food frequency questionnaire.

Food Groups (Consumption)			Measurement Time			
			Pre-Intervention *n* (%)	Post-Intervention *n* (%)	*p*-Value ^+^	*p*-Value *
Fruits	Control	Never/rarely	4 (12.5)	3 (13.0)	0.730	0.428
		Weekly	13 (40.6)	7 (30.4)		
		Daily	15 (46.9)	13 (56.5)		
	Intervention	Never/rarely	4 (12.9)	2 (8.3)	0.674	
		Weekly	15 (48.4)	10 (41.7)		
		Daily	12 (38.7)	12 (50.0)		
Vegetables	Control	Never/rarely	2 (6.3)	1 (4.3)	0.932	0.088
		Weekly	9 (28.1)	6 (26.1)		
		Daily	21 (65.6)	16 (69.6)		
	Intervention	Never/rarely	3 (9.7)	0 (0.0)	0.254	
		Weekly	5 (16.1)	3 (12.5)		
		Daily	23 (74.2)	21 (87.5)		
Plant-based protein	Control	Never/rarely	16 (50)	11 (47.8)	0.856	0.203
		Weekly	12 (37.5)	10 (43.5)		
		Daily	4 (12.5)	2 (8.7)	0.174	
	Intervention	Never/rarely	9 (29.0)	6 (25.5)		
		Weekly	14 (45.2)	16 (66.7)		
		Daily	8 (25.8)	2 (8.3)		
Wholegrains	Control	Never/rarely	21 (65.6)	15 (65.2)	0.737	0.735
		Weekly	8 (25.0)	7 (30.4)		
		Daily	3 (9.4)	1 (4.3)		
	Intervention	Never/rarely	15 (48.4)	13 (54.2)	0.038	
		Weekly	9 (29.0)	11 (45.8)		
		Daily	7 (22.6)	0 (0.0)		
Nuts & Seeds	Control	Never/rarely	11 (34.4)	7 (30.4)	0.222	0.736
		Weekly	15 (46.9)	7 (30.4)		
		Daily	6 (18.8)	9 (39.1)		
	Intervention	Never/rarely	9 (29.0)	5 (20.8)	0.608	
		Weekly	14 (45.2)	10 (41.7)		
		Daily	8 (25.8)	9 (37.5)		
Probiotics	Control	Never/rarely	2 (6.3)	3 (13.0)	0.649	0.996
		Weekly	15 (46.9)	9 (39.1)		
		Daily	15 (46.9)	11 (47.8)		
	Intervention	Never/rarely	4 (12.9)	3 (12.5)	0.958	
		Weekly	13 (41.9)	11 (45.8)		
		Daily	14 (45.2)	10 (41.7)		
Herbs	Control	Never/rarely	15 (46.9)	12 (52.2)	0.899	0.639
		Weekly	7 (21.9)	4 (17.4)		
		Daily	10 (31.3)	7 (30.4)		
	Intervention	Never/rarely	22 (71.0)	11 (45.8)	0.087	
		Weekly	5 (16.1)	4 (16.7)		
		Daily	4 (12.9)	9 (37.5)		
MUFA	Control	Never/rarely	21 (65.6)	5 (21.7)	0.005	0.962
		Weekly	3 (9.4)	4 (17.4)		
		Daily	8 (25.0)	14 (60.9)		
	Intervention	Never/rarely	25 (80.6)	9 (37.5)	0.003	
		Weekly	4 (12.9)	6 (25.0)		
		Daily	2 (6.5)	9 (37.5)		
PUFA	Control	Never/rarely	2 (6.3)	1 (4.3)	0.296	0.996
		Weekly	3 (9.4)	0 (0.0)		
	Intervention	Daily	27 (84.4)	22 (95.7)	0.063	
		Never/rarely	2 (6.5)	1 (4.2)		
		Weekly	6 (19.4)	0 (0.0)		
		Daily	23 (74.2)	23 (95.8)		
Animal-based protein	Control	1–3/day	29 (90.6)	22 (100)	0.200	0.858
		4+/day	3 (9.4)	0 (0.0)		
	Intervention	1–3/day	31 (100)	18 (78.3)	0.011	
		4+/day	0 (0.0)	5 (21.7)		

Pearson’s chi-square was used for all the food groups except for animal-based protein (*p*-value < 0.05 is considered statistically significant). Fisher’s exact test was used for animal-based protein (*p*-value < 0.05 is considered statistically significant). MUFA; monounsaturated fatty acids, PUFA; polyunsaturated fatty acids, *n*; sample size. ^+^: Comparison within groups using Pearson’s chi-square test. *: Comparison between groups using multinomial logistic regression of post-intervention measurements versus pre-intervention measurements and groups while controlling for educational level and marital status.

**Table 4:** Intake of immune-enhancing nutrients for both groups pre- versus post-intervention—relative values.

	Control Group		Intervention Group		*p*-Value (ITT)	Effect Size ^§^ (ITT)
Variable	Mean ± SD	(Min–Max) at Baseline	Mean ± SD	(Min–Max) at Baseline		
Iron (mg)_pre (*n* = 62)	13 ± 7.5	4.98–42.93	14 ± 8.18	3.6–35.26	0.586	0.0023
Iron (mg)_post (*n* = 47)	15 ± 9.12		13 ± 7.45			
Vitamin C (mg)_pre (*n* = 62)	92 ± 132.37	8.12–446.12	38 ± 33.64	0.00–143.63	0.804	0.0001
Vitamin C (mg)_post (*n* = 47)	91 ± 155.17		42 ± 19.39			
Vitamin D-IU (IU)_pre (*n* = 62)	50 ± 147.85	0.00–819.71	41 ± 69.37	0.00–356.01	0.624	0.0072
Vitamin D-IU (IU)_post (*n* = 46)	54 ± 95.65		26 ± 51.17			
Vitamin A RAE (mcg)_pre (*n* = 61)	1365 ± 2219.71	0.00–5539.02	1289 ± 2891.31	0.00–12,509.12	0.439	0.0100
Vitamin A RAE (mcg)_post (*n* = 46)	2725 ± 8253.43		1467 ± 2229.06			
Zinc (mg)_pre (*n* = 62)	7 ± 4.41	1.97–23.37	7 ± 4.88	1.21–24.73	0.888	0.0015
Zinc (mg)_post (*n* = 47)	5 ± 2.48		7 ± 4.52			
Vitamin E-mg (mg)_pre (*n* = 53)	1 ± 1.33	0.06–4.22	0.6 ± 0.76	0.01–3.52	0.042	0.0343
Vitamin E-mg (mg)_post (*n* = 40)	0.51 ± 0.59		0.8 ± 0.91			
Omega 3 Fatty Acid (g)_pre (*n* = 59)	0.34 ± 0.48	0.02–2.27	0.62 ± 0.99	0.03–5.15	0.946	0.0053
Omega 3 Fatty Acid (g)_post (*n* = 46)	0.41 ± 0.66		0.7 ± 0.8			
Omega 6 Fatty Acid (g)_pre (*n* = 59)	6 ± 17.48	0.07–95.37	4 ± 4.11	0.10–16.23	0.779	0.0003
Omega 6 Fatty Acid (g)_post (*n* = 46)	4 ± 4.1		5 ± 5.89			
Selenium (mcg)_pre (*n* = 62)	58 ± 62.55	3.04–306.14	87 ± 61.31	4.51–316.94	0.959	0.0036
Selenium (mcg)_post (*n* = 46)	32 ± 34.54		87 ± 85.48			
Protein (g)_pre (*n* = 62)	100 ± 46.88	51.25–220.4	105 ± 52.31	39.51–271.07	0.646	0.0018
Protein (g)_post (*n* = 47)	103 ± 50.48		101 ± 53.05			
Calories (kcal)_pre (*n* = 62)	2131 ± 929.63	1061.96–5401.83	2210 ± 1351.68	726.06–6530.22	-	0.0150
Calories (kcal)_post (*n* = 47)	2379 ± 970.95		2069 ± 965.43			
Calcium (mg)_pre (*n* = 62)	561 ± 307.75	95.78–1435.47	465 ± 311.06	66.09–1085.16	-	0.0014
Calcium (mg)_post (*n* = 47)	597 ± 361.83		583 ± 383.36			

Linear regression was used to compare the mean change in value from pre- to post-intervention between the control and the intervention group. (*p*-value < 0.05 is statistically significant) while controlling for educational level and marital status. EPA: eicosapentaenoic acid, DHA: docosahexaenoic acid, mg: milligrams, mcg: micrograms, g: grams, IU: international unit, Kcal: kilocalories, PP: per-protocol, ITT: intention-to-treat, SD: standard deviation. The sample size differed based on the method of analysis, with 63 participants for the ITT analysis and 46 for the PP analysis. -: ITT analysis was performed only on immune-enhancing nutrients that have been shown in the literature to impact the overall health and immunity of PLHIV. ^§^: Cohen’s f-square measure of effect size. Interpretation: 0.02 (small effect), 0.15 (medium), 0.35 (large) [20].

## Data Availability

Due to ethical considerations, the authors cannot make any additional interview data publicly available because they contain potentially identifiable and confidential patient information, with the possibility that individuals may be recognized in the data. Additional data may be shared upon reasonable request to the Mohammed Bin Rashed University Research Ethics Committee; email: irb@dubaihealth.ae.

## Supplementary Materials

The following supporting information can be downloaded at https://www.mdpi.com/article/10.3390/nu18111709/s1, Supplementary Material S1: CONSORT 2025 checklist [44].

## Abbreviations

The following abbreviations are used in this manuscript:

DSREC	Dubai Scientific Research Ethics Committee
FFQ	Food Frequency Questionnaire
HBM	Health Belief Model
KAP	Knowledge, Attitude, and Practices
MENA	Middle East and North Africa
MUFA	Monounsaturated Fatty Acid(s)
PP	Per-Protocol
